# Supplementary material for: HetL, HetR and PatS form a reaction-diffusion system to control pattern formation in the cyanobacterium nostoc PCC 7120
Source: eLife. 2020 Aug 7;9:e59190. doi: 10.7554/eLife.59190 (PMC7476756; doi:10.7554/eLife.59190)
Supplement: Supplementary file 1. — Sequences of the primers used. Supplementary Table 1. PRPs encoding genes in the genome of Nostoc The sequences were retrieved from the Microscope Mage database. The genes located on Nostoc plasmids are marked by an asterisk. The functional domains were analyzed by Pfamscan. The cellular localization was deduced from the presence of transmembrane domain, signal peptide and lipopeptide using TMhmm, SignalIP and LipoP softwires. For gene expression, the reads values are those reported in Flaherty et al., 2011. Supplementary Table 2. List of active residues used as input for HetRhood:HetL docking simulations [file elife-59190-supp1.docx]

**Supplementary file 1**

***Nostoc* and *E. coli* strains**

| **Strains** | **Description/genotype** | **Source/reference** |
| --- | --- | --- |
| ***Nostoc* strains** |  |  |
| *Nostoc* PCC 7120 | Wild type strain (WT) | Pasteur Institute Collection |
| WT/P*petE-patS* | WT strain containing the pRL1272-P*petE-patS* plasmid | This study |
| WT/P*petE-patS* P*petE-hetL* | WT strain containing the pRL1272-P*petE-patS* plasmid and pRL25T-P*petE-hetL* plasmid | This study |
| WT/P*petE-patS* P*petE-hetL*D151A | WT strain containing the pRL1272-P*petE-patS* plasmid and pRL25T-P*petE-hetL*D151A plasmid derivative where the *hetL* gene has been mutated to encode for a D151A substitution | This study |
| WT/P*petE-hetL* | WT strain containing the pRL25T-P*petE-hetL* plasmid | This study |
| WT/P*petE-hetL*D151A | WT strain containing the pRL25T-P*petE-hetL*D151A plasmid | This study |
| WT/P*patS-hetL* | WT strain containing the pRL25T-P*patS-hetL* plasmid | This study |
| WT/P*rbcL-hetL* | WT strain containing the pRL25T-P*rbcL-hetL* plasmid | This study |
| Δ*hetR* | *Nostoc* deletion mutant of the *hetR* gene | (Borthakur, Orozco, Young-Robbins, Haselkorn, & Callahan, 2005) |
|  |  |  |
| ***E. coli* strains** |  |  |
| TG1 | *supE thi*-1 ∆(*lac-proAB*) ∆(*mcrB-hsdSM*)5 (rK– mK–) [F´ *traD36 proAB lacIqZ∆M15*] | Stratagene |
| DH5α | *fhuA2 lac(del)U169 phoA glnV44 Φ80' lacZ(del)M15 gyrA96 recA1 relA1 endA1 thi-1 hsdR17* | (Taylor, Walker, & McInnes, 1993) |
| Stellar^TM^ | *F–, endA1, supE44, thi-1, recA1, relA1, gyrA96, phoA, Φ80d lacZΔ M15, Δ (lacZYA - argF) U169, Δ (mrr - hsdRMS - mcrBC), ΔmcrA, λ–* | Takara |
| BL21DE3 | *fhuA2 [lon] ompT gal (λ DE3) [dcm] ∆hsdS λDE3 = λsBamHIo ∆EcoRI-int::(lacI::PlacUV5::T7 gene1) i21∆nin5* | NEB |
| BTH101 | F^−^, cya-99, araD139, galE15, galK16, rpsL1, hsdR2, mcrA1, mcrB1 | (Karimova et al., 1998) |
| eXX1 | TG1 containing the plasmid pKT25-*hetR* | This study |
| eXX2 | TG1 containing the plasmid pKT25-*hetL* | This study |
| eXX3 | TG1 containing the plasmid pKT25-*hetL*D151A | This study |
| eXX4 | TG1 containing the plasmid pUT18C-*hetR* | This study |
| eXX5 | TG1 containing the plasmid pUT18C-*hetR*R223W | This study |
| eXX6 | TG1 containing the plasmid pUT18C-*hetL* | This study |
| eXX7 | TG1 containing the plasmid pUT18C-*hetL* RBS*-patS* | This study |
| eXX8 | TG1 containing the plasmid *hetR*_hood_-pUT18 | This study |
| eXX9 | TG1 containing the plasmid pUT18C-all3256 | This study |
| eXX10 | TG1 containing the plasmid pUT18C-all4303 | This study |
| eBR1 | TG1 containing the plasmid pET28a-*his-hetR* | (Roumezi et al., 2019) |
| eXX11 | Stellar^TM^ containing the plasmid pRL1272-P*petE-patS* | This study |
| eXX12 | Stellar^TM^ containing the plasmid pRL25T-P*petE-hetL* | This study |
| eXX13 | Stellar^TM^ containing the plasmid pRL25T-P*petE-hetL*D151A | This study |
| eSC1 | Stellar^TM^ containing the plasmid pRL25T-P*patS-hetL* | This study |
| eSC2 | Stellar^TM^ containing the plasmid pRL25T-P*rbcL-hetL* | This study |
| eXX14 | BTH101 containing plasmids pKT25-zip and pUT18C-zip | This study |
| eXX15 | BTH101 containing plasmids pKT25 and pUT18C | This study |
| eXX16 | BTH101 containing plasmids pKT25-*hetL* and pUT18C | This study |
| eXX17 | BTH101 containing plasmids pKT25 and pUT18C-*hetR* | This study |
| eXX18 | BTH101 containing plasmids pKT25-*hetR* and pUT18C-*hetR* | This study |
| eXX19 | BTH101 containing plasmids pKT25-*hetL* and pUT18C-*hetR* | This study |
| eXX20 | BTH101 containing plasmids pKT25-*hetL* and pUT18C-*hetL* | This study |
| eXX21 | BTH101 containing plasmids pKT25-*hetL* and *hetR*_hood_-pUT18 | This study |
| eXX22 | BTH101 containing plasmids pKT25-*hetL*D151A and pUT18C-*hetL* | This study |
| eXX23 | BTH101 containing plasmids pKT25-*hetR* and pUT18C-*hetR*R223W | This study |
| eXX24 | BTH101 containing plasmids pKT25-*hetL*D151A and pUT18C-*hetR* | This study |
| eXX25 | BTH101 containing plasmids pKT25-*hetL* and pUT18C-*hetR*R223W | This study |
| eXX26 | BTH101 containing plasmids pKT25-*hetR* and pUT18C-*hetL* | This study |
| eXX27 | BTH101 containing plasmids pKT25-*hetR* and pUT18C-*hetL-*RBS*-patS* | This study |
| eXX28 | BTH101 containing plasmids pKT25-*hetR* and pUT18C-*hetL-*RBS*-patS6* | This study |
| eXX29 | BTH101 containing plasmids pKT25-*hetR* and pUT18C-all3256 | This study |
| eXX30 | BTH101 containing plasmids pKT25-*hetR* and pUT18C-all4303 | This study |

**Plasmids**

| **Plasmids** | **Description** | **Source/reference** |
| --- | --- | --- |
| pKT25-zip | Two hybrid plasmid Kan^R^ | (Karimova et al., 1998) |
| pUT18C-zip | Two hybrid plasmid Amp^R^ | (Karimova et al., 1998) |
| pKT25 | Two hybrid plasmid. T25 at the N terminus Kan^R^ | (Karimova et al., 1998) |
| pUT18C | Two hybrid plasmid. T18 at the N terminus Amp^R^ | (Karimova et al., 1998) |
| pKNT25 | Two hybrid plasmid. T25 at the C terminus Kan^R^ | (Karimova et al., 1998) |
| pUT18 | Two hybrid plasmid. T18 at the C terminus. Amp^R^ | (Karimova et al., 1998) |
| pET28a | His-tagged protein expression plasmid in *E. Coli* Kan^R^ | Novagen |
| pRL1272 | Replicative in *Nostoc* Ery^R^ | (Wolk et al., 1988) |
| pRL25T | Replicative in *Nostoc* Neo^R^ | (Yang et al., 2013) |
| pXX1 | pKT25-*hetR* | This study |
| pXX2 | pKT25-*hetL* | This study |
| pXX3 | pKT25-*hetL*D151A | This study |
| pXX4 | pUT18C-*hetR* | This study |
| pXX5 | pUT18C-*hetR*R223W | This study |
| pXX6 | pUT18C-*hetL* | This study |
| pXX7 | pUT18C-*hetL-*RBS*-patS* | This study |
| pXX8 | pUT18C-*hetL-*RBS*-patS6* | This study |
| pXX9 | *hetR*_hood_-pUT18 | This study |
| pXX10 | pUT18C-all3256 | This study |
| pXX11 | pUT18C-all4303 | This study |
| pBR1 | pET28a-*his-hetR* | (Roumezi et al., 2019) |
| pXX12 | pET28a-*hetL-his* | This study |
| pCSB270 | pRL1272-P*petE* | Lab collection |
| pXX13 | pRL1272-P*petE-patS* | This study |
| pCSB265 | pRL25T-P*petE* | Lab collection |
| pXX14 | pRL25T-P*petE-hetL* | This study |
| pXX15 | pRL25T-P*petE-hetL*D151A | This study |
| pSC1 | pRL25T-P*patS-hetL* | This study |
| pSC2 | pRL25T-P*rbcL-hetL* | This study |

**Primers**

| Name | Sequence (5’-3’) | Experiment | |
| --- | --- | --- | --- |
| 16S rRNA rt fw | TCCTGGTGTAGCGGTGAAAT | Quantitative RT-PCR analysis | |
| 16S rRNA rt rv | AGCCACGCCTAGTATCCATC |  |  |
| *hetP* rt fw | TGGCTGGTAAATACTCTTGGG |  |  |
| *hetP* rt rv | ACCTACTACTTCCAGATAGGC |  |  |
| *hetL* rt fw | GACATTATGCTGCTGGCAAA |  |  |
| *hetL* rt rv | CAAGTCGCGTCTGACGTAAA |  |  |
| *hetR* rt fw | GCGTCGTCTGCTTTACTCTG |  |  |
| *hetR* rt rv | CCCAGTCTTTCATCATGCGG |  |  |
| *hetR* dh fw T25 | TTTTCTGCAGGGATGAGTAACGACATCGATCT |  |  |
| *hetR* dh fw T18 | TTTTCTGCAGGATGAGTAACGACATCGATCTGA |  |  |
| *hetR* dh rv | TTTTTGAATTCTTAATCTTCTTTTCTACCAAACACCATTTG | Two hybrid assays |  |
| Mut *hetR*R223W fw | CCAGCAGACGACCAAGAGTGGACTTATATTATGGTGGAA |  |  |
| Mut *hetR*R223W rv | TTCCACCATAATATAAGTCCACTCTTGGTCGTCTGCTGG |  |  |
| *hetL* dh fw T25 | TTTTCTGCAGGGATGAATGTGGGTGAAAT |  |  |
| *hetL* dh fw T18 | TTTTCTGCAGGATGAATGTGGGTGAAAT |  |  |
| *hetL* dh rv | TTTTTGAATTCTCAATCATGAATTGAACCATCAGG |  |  |
| RBS*-patS dh* fw T18 | TTTTTGTCGACAGGTTAGGAGAACCATATG |  |  |
| RBS*-patS dh* rv T18 | TTTTTCTCGAGGATTGAGTGGTCGGAACGA |  |  |
| RBS*-patS6 dh* fw T18 | AAGCTTATCGATACCGTCGACAGGTTAGGAGAACCATATGGAGCGCGGTAGTGGTAGATAGAACG |  |  |
| RBS*-patS6 dh* rv T18 | GATGAATTGCTCGAGGTCGACGATTGAGTGGTCGGAACGAATGC |  |  |
| *hetR*_hood_ dh fw | TTTTCTGCAGGTATGCCCCAGCAGAC |  |  |
| *hetR*_hood_ dh rv | TTTTTGAATTCGATCTACCAAACACCATTTGTAAAATCATGG |  |  |
| all3256 dh fw T18 | TTTTCTGCAGGATGGCAAATCTAGAGCAT |  |  |
| all3256 dh rv | TTTTTGAATTCCTAATCATGCCTTGAAGAGTCA |  |  |
| all4303 dh fw T18 | TTTTCTGCAGGATGAATATTGACGCTATT |  |  |
| all4303 dh rv | TTTTTGAATTCTTACCCATTACCAATTTCTAATATTGTCCCT |  |  |
| Mut *hetL*D151A fw | GCAGATTTAAGCTACGCTGCCCTGAGAGCGGCTTCTCTA |  |  |
| Mut *hetL*D151A rv | TAGAGAAGCCGCTCTCAGGGCAGCGTAGCTTAAATCTGC |  |  |
| *hetR* pET28 fw | CATATGAGTAACGACATCGATCTG |  |  |
| *hetR* pET28 rv | GGATCCTTAATCTTCTTTTCTACC |  |  |
| *hetL* pET28 fw | AGGAGATATACCATGGGCAATGTGGGTGAAATTCTGAGACA | Protein production for BLI assays |  |
| *hetL* pET28 rv | GGTGGTGGTGCTCGAGACCTTGAAAATAAAGATTTTCATCATGAATTGAACCATCA |  |  |
| *patS* pRL fw | GCCCATCGATGGATCCATGAAGGCAATTATGTTAGTG |  |  |
| *patS* pRL rv | CGTCGACCCGGGATCCATGACTATTGACCAAATGACTATTG |  |  |
| *hetL* pRL fw | GAGCTCGTCGACCCGGGATCCTCAATCATGAATTGAACCATCAGGC | Construction of recombinant plasmids for *Nostoc* |  |
| *hetL* pRL rv | ATGGGGCCCATCGATGGATCCATGAATGTGGGTGAAATTCTGAGAC |  |  |
| P*patS* fw | TGAGATTATCAAAAAGGATCCAGATCC  TGAATTTGTTTTGGGAAC |  |  |
| P*patS* rv | CCACATTCATAATCTTAACCTCCCTGAATTACTTTTCAACAGAACATTT |  |  |
| *hetL* P*patS* fw | TAAGATTATGAATGTGGGTGAAATTCT  GAGAC |  |  |
| *hetL* P*patS* rv | GAGTAGAATTCCCGGGGATCCTCAATC  ATGAATTGAACCATCAGGC |  |  |
| P*rbcL* fw | TGAGATTATCAAAAAGGATCCGCAGGGGAAGTAAAGAAGAATGAC |  |  |
| P*rbcL* rv | CACCCACATTCATATCTATCCTTCCAAGATGTCAC |  |  |
| *hetL* P*rbcL* fw | GATAGATATGAATGTGGGTGAAATTCTGAGAC |  |  |
| *hetL* P*rbcL* rv | GAGTAGAATTCCCGGGGATCCTCAATCATGAATTGAACCATCAGGC |  |  |
| P*hetP* fw | [6FAM]ATTTAGTGGTAAATTCTCTT | EMSA assay |  |
| P*hetP* rv | TGAGTTATACGCTATATCAA |  |  |

**Supplementary Table 1: PRPs encoding genes in the genome of *Nostoc***

| **Gene ID/name** | **Num**  **of**  **AA** | **Num**  **of PRs domains** | **Additional**  **Domain(s)** | **Cellular**  **location** | **Gene expression (number of reads)**  **0h 6h 12h 21h** |
| --- | --- | --- | --- | --- | --- |
| **Genes whose expression changes in response to combined nitrogen starvation** | | | | | |
| alr4610 | 164 | 2 | - | P/L | 33 30 88 92 |
| alr1298 | 167 | 1 | - | C | 79 65 151 119 |
| all0186 | 168 | 2 | - | M | 13 6 74 30 |
| alr1746 | 182 | 3 | - | C | 843  519 134 159 |
| all4303 | 213 | 4 | - | C | 39 51 33 80 |
| all3048 | 217 | 2 | DnaJ | C | 96 84 131 195 |
| all2395 (FraF) | 222 | 3 | - | C | 15 11 105 80 |
| alr1579 | 222 | 3 | - | P/L | 4 5 67 8 |
| all3740 (HetL) | 237 | 4 | - | C | 16 18 50 78 |
| all4152 | 450 | 1 | - | M | 9 21 28 27 |
| all3305(PatL) | 496 | 5 | - | M | 76 94 163 137 |
| alr3268 | 524 | 2 | Kinase | C | 6 8 17 23 |
| all3114 | 576 | 5 | - | C | 19 17 56 68 |
| alr9014* | 679 | 3 | - | M | 40 40 87 73 |
| alr0704 | 693 | 3 | - | M | 21 28 39 42 |
| all0813 (HglK) | 727 | 4 | RDD domain | M | 65 52 316 222 |
| **Genes whose expression is not impacted by combined nitrogen starvation** | | | | | |
| all1812 | 125 | 1 | - | C |  |
| alr5209 | 129 | 2 | - | C |  |
| alr0433 | 143 | 2 | - | P/L |  |
| all4220 | 152 | 1 | - | P/L |  |
| alr2741 | 182 | 2 | - | M |  |
| all3332 | 206 | 1 |  | M |  |
| all3306 | 252 | 2 | - | C |  |
| alr2768 | 256 | 1 | - | L/P |  |
| all3256 | 268 | 4 | - | C |  |
| alr7125* | 369 | 2 | - | C |  |
| all3869 | 376 | 4 | Endoribonuclease L-PSP | C |  |
| all0958 | 475 | 2 | - | C |  |
| alr1142 | 521 | 3 | Pentapeptide 4 (9PRs) | C |  |
| alr7124* | 586 | 2 | - | M |  |
| alr3131 | 953 | 2 | - | C |  |
| all8023 | 1010 | 3 | - | C |  |

**Supplementary table 2**

**List of active residues used as input for HetRhood:HetL docking simulations**

| ***Protein*** | ***Active residues number*** |
| --- | --- |
| HetL | 0,1,2,3,5,7,8,9,10,11,12,13,14,15,16,18,19,20,21,23,24,26,28,29,31,33,36,39,43,44,46,49,51,53,54,58,59,61,64,66,68,71,74,78,79,81,83,84,88,89,91,93,94,96,98,99,103,104,106,108,111,113,114,116,119,121,123,124,126,128,129,130,131,132,133,134,136,139,141,143,144,146,149,151,153,158,159,161,163,164,166,169,171,174,175,176,177,178,179,180,181,184,186,187,189,191,192,194,197,199,201,204,206,207,209,211,212,214,215,216,217,218,219,220,221,222,224,225,226,227,228,230,231,232,233,234,235,236 |
| HetR hood | 222,223,224,225,227,228,229,231,233,235,236,239,240,241,243,244,245,246,247,249,250,252,253,254,256,259,260,261,262,263,264,266,267,268,269,271,272,273,274,276,279,280,281,282,283,285,287,296,297,298 |
